# Artificial intelligence-driven prognostic system for conception prediction and management in intrauterine adhesions following hysteroscopic adhesiolysis: a diagnostic study using hysteroscopic images

**DOI:** 10.3389/fbioe.2024.1327207

**Published:** 2024-04-04

**Authors:** Bohan Li, Hui Chen, Hua Duan

**Affiliations:** ^1^ Department of Minimally Invasive Gynecologic Center, Beijing Obstetrics and Gynecology Hospital, Capital Medical University, Beijing Maternal and Child Healthcare Hospital, Beijing, China; ^2^ School of Biomedical Engineering, Capital Medical University, Beijing, China; ^3^ Beijing Advanced Innovation Center for Big Data-based Precision Medicine, Capital Medical University, Beijing, China

**Keywords:** intrauterine adhesions, Cox proportional hazard network, image deep learning, treatment recommendation, subfertility

## Abstract

**Introduction:**

Intrauterine adhesions (IUAs) caused by endometrial injury, commonly occurring in developing countries, can lead to subfertility. This study aimed to develop and evaluate a DeepSurv architecture-based artificial intelligence (AI) system for predicting fertility outcomes after hysteroscopic adhesiolysis.

**Methods:**

This diagnostic study included 555 intrauterine adhesions (IUAs) treated with hysteroscopic adhesiolysis with 4,922 second-look hysteroscopic images from a prospective clinical database (IUADB, NCT05381376) with a minimum of 2 years of follow-up. These patients were randomly divided into training, validation, and test groups for model development, tuning, and external validation. Four transfer learning models were built using the DeepSurv architecture and a code-free AI application for pregnancy prediction was also developed. The primary outcome was the model’s ability to predict pregnancy within a year after adhesiolysis. Secondary outcomes were model performance which evaluated using time-dependent area under the curves (AUCs) and C-index, and ART benefits evaluated by hazard ratio (HR) among different risk groups.

**Results:**

External validation revealed that using the DeepSurv architecture, InceptionV3+ DeepSurv, InceptionResNetV2+ DeepSurv, and ResNet50+ DeepSurv achieved AUCs of 0.94, 0.95, and 0.93, respectively, for one-year pregnancy prediction, outperforming other models and clinical score systems. A code-free AI application was developed to identify candidates for ART. Patients with lower natural conception probability indicated by the application had a higher ART benefit hazard ratio (HR) of 3.13 (95% CI: 1.22–8.02, *p* = 0.017).

**Conclusion:**

InceptionV3+ DeepSurv, InceptionResNetV2+ DeepSurv, and ResNet50+ DeepSurv show potential in predicting the fertility outcomes of IUAs after hysteroscopic adhesiolysis. The code-free AI application based on the DeepSurv architecture facilitates personalized therapy following hysteroscopic adhesiolysis.

## 1 Introduction

Intrauterine adhesions (IUAs), commonly known as Asherman’s syndrome, are caused by injury to the endometrial basal layer and subsequent scar formation (Xu et al., 2018). IUAs are more common in developing countries, often secondary to induced abortions or intrauterine procedures, with incidence rates of up to 14% in patients with infertility or recurrent pregnancy loss (RPL) (Bosteels et al., 2018; Zhao et al., 2021). Hysteroscopic adhesiolysis is the current therapy for IUAs due to its minimally invasive nature and direct visualization (Yu et al., 2008; Hooker et al., 2014; Hanstede et al., 2021). However, managing moderate to severe IUAs remains challenging, and severe cases are associated with poor prognoses (Zhao et al., 2021).

The main methods for evaluating the prognosis of IUA patients are hysteroscopy and ultrasound. Hysteroscopy can comprehensively analyze postoperative uterine cavity morphology and endometrial receptivity (Smit et al., 2016; Craciunas et al., 2019), thus becoming the first-line assessment method in clinical practice. Moreover, some studies have shown that postoperative hysteroscopic assessment is more powerful in predicting the fertility outcomes of patients (Xu et al., 2018; Zhao et al., 2022). However, without objective indicators, it has difficulties effectively integrating it with ART management for postoperative patients. Artificial intelligence (AI) algorithms can provide objective assessment metrics for endoscopic techniques (Zhang et al., 2021; Sutton et al., 2022), which has gradually received attention in the field of IUAs. In our previous study, we used machine learning algorithms such as decision trees and XGboost to predict the pregnancy outcomes of IUA patients based on clinical data (Zhu et al., 2022; Li et al., 2023a). Recently, some studies have also used 3D ultrasound parameters to predict the outcomes (Sun et al., 2024). These studies have achieved good prediction results, but they have some limitations. The data they used are based on the clinical scores and manual measurements by doctors, which may have subjectivity and heterogeneity issues. Moreover, the lack of external validation makes these algorithms challenging to apply in clinical practice (Zhu et al., 2022; Sun et al., 2024).

However, there is currently a lack of research on image deep learning for hysteroscopy in IUA, which can automatically extract and analyze features from hysteroscopic images and achieve objectivity and accuracy to some extent. Compared with other fields, hysteroscopic image deep learning for IUA faces some challenges due to the lack of previous research, such as determining the optimal angle of images, standardizing the process, selecting a model that can balance the computational resources and accuracy, and adjusting the training parameters. In addition, predicting fertility outcomes is essentially a risk prognosis task involving both the time and cumulative hazard of event occurrence. Therefore, choosing a suitable model to extract features from hysteroscopic images and to predict the prognosis events is very important.

Transfer learning models based on Convolutional Neural Networks (CNN) indicate potential in medical image analysis (Esteva et al., 2019; Ling et al., 2022). In this study, we employ three CNN architectures for hysteroscopic image classification: InceptionV3, ResNet50, and InceptionResNetV2. InceptionV3 utilizes multiple convolutional filters of varying sizes to extract features at different scales, providing a wider receptive field (Szegedy et al., 2016). ResNet50 incorporates residual connections to alleviate the vanishing gradient problem and improve training stability (He et al., 2016). InceptionResNetV2 combines the strengths of both Inception and ResNet, offering a deeper and more efficient architecture for image recognition (Szegedy et al., 2017). These models have demonstrated state-of-the-art performance in various image classification tasks, including medical image analysis. The evaluation of various CNN transfer learning models in hysteroscopy may reveal their strengths and limitations for IUA fertility outcomes prediction.

On top of the transfer learning framework, we further attempt to add DeepSurv, which is a feed-forward neural network based on the Cox proportional hazards (CPH) model, to assess the risk of event occurrence. CPH is a commonly used semi-parametric model in medical statistics to measure the effect of covariates on the hazard of event occurrence, which enables the prediction of time-to-event outcomes. DeepSurv extends CPH to the application of non-linear parameters, which can be used for image feature analysis, and thus achieve medical prognosis stratification and management (Katzman et al., 2018; Katzman et al., 2018; She et al., 2020).

As an overview, despite the prevalence of IUAs in developing countries, there is a lack of objective and accurate methods to predict fertility outcomes after surgery. This gap hinders personalized treatment planning and optimization of patient care. Therefore, we hypothesize that transfer learning models with DeepSurv architecture can predict the reproductive prognosis in IUA patients by hysteroscopic image and suggest stratified therapy following hysteroscopic adhesiolysis. This approach aims to contribute to personalized diagnosis and treatment for patients by filling the research gap in the field of IUA with AI techniques.

## 2 Materials and methods

### 2.1 Study design and participants

**FIGURE 1:**
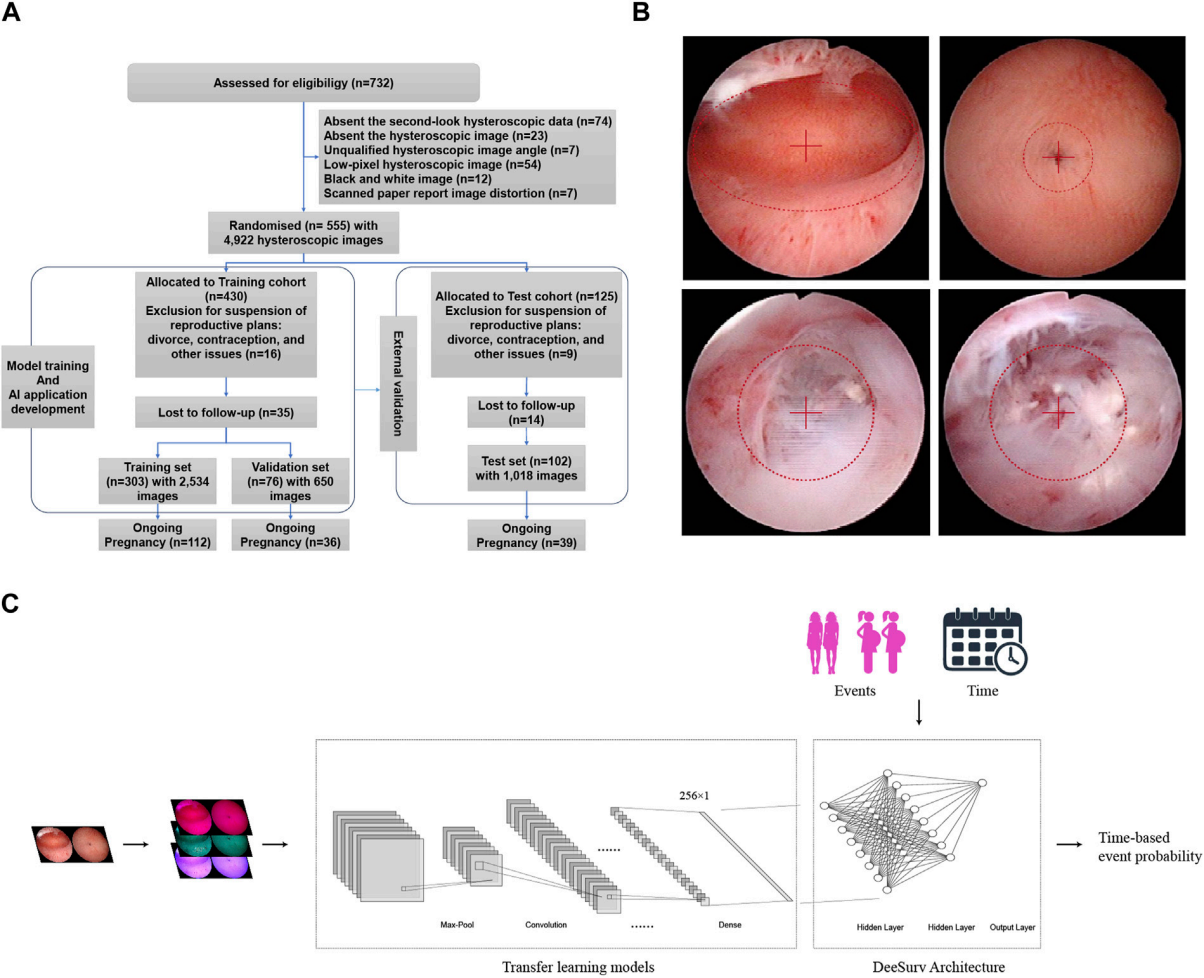
**(A)** Flow diagram of the cohort study. **(B)** Example of standardized image acquisition. Ensure the hysteroscope lens is level when capturing images of the uterine cavity, positioning the center point at the visible uterine cavity’s midpoint. For images of both uterine corners, place the center point at the fallopian tube orifice. If there are adhesions closing the fallopian tube orifice, center the adhesion site in the field of view. **(C)** Models block diagram of transfer learning models + DeepSurv.

Data were obtained from the Intrauterine Adhesion Prospective Clinical Database (ClinicalTrials.gov NCT05381376), which enrolled 732 patients prospectively between December 2018 and January 2020. Inclusion criteria are as follows: i) hysteroscopy-confirmed IUA; ii) recent plans for conception; and iii) normal hormone levels and ovulation. Exclusion criteria included i) male infertility, ii) primary infertility, iii) tuberculosis-related IUA, iv) tubal factor infertility, and v) other diseases such as endometrial polyps, atypical hyperplasia, or endometrial cancers. The study included 555 patients, who were randomly assigned in a 3:1 ratio to the modeling cohort or the test cohort. The modeling cohort consisted of 430 patients, who were further randomly divided into training and validation sets in an 8:2 ratio using computer-based randomization. The training and validation sets were used for model training and hyperparameter tuning, respectively. The test cohort consisted of 125 patients, who were used for external validation of the model (Figure 1A). Following a thorough explanation of the procedure, informed consent was obtained.

### 2.2 Hysteroscopic adhesiolysis and postoperative management

Twelve hours before surgery, 1 mg of derivative of prostaglandin F2α (PGF2α) was administered for cervical softening. Surgical hysteroscopy was performed and normal saline as the perfusion medium. The shape and extent of adhesion in the uterine cavity were assessed directly. The endometrium was preserved by performing meticulous adhesion tissue separation and scar tissue removal. Successful separation required the restoration of normal uterine anatomy without adhesions. Physical barriers were used postoperatively to prevent adhesion recurrence, followed by a three-month course of estradiol valerate and dydrogesterone-based hormonal therapy. Patients were encouraged to attempt natural conception for a year after surgery, with ART treatment indicated if pregnancy did not occur within that timeframe (Li et al., 2024).

### 2.3 Data collection and pre-processing

Following Standard Operating Procedures (SOPs), 4,922 postoperative hysteroscopic follow-up images were obtained 3–7 days after menstruation. Using 0.9% saline for irrigation, specialized hysteroscopy physicians maintained uterine distension at 80–100 mmHg pressure with a flow rate of 260–280 mL/min for optimal fallopian tube ostia visualization. Intrauterine orthoimages and fallopian tubes ensured clear images centered within the lens (Figure 1B). Supplementary Table S1 describes the image collection process. Age, endometrial thickness, endometrial blood supply, fallopian tube ostia, and the clinical scoring systems AFS (American Fertility Society) and CSGE (Chinese Society of Gynaecological Endoscopy) (Supplementary Table S2) were collected as clinical data. Reproductive outcomes were assessed using medical records and telephonic follow-ups at least 2 years after the intervention. Study endpoints included continued pregnancy, defined as detecting a fetal heartbeat at >12 weeks of gestation (Smit et al., 2016).

The study followed the STARD 2015 guidelines (Bossuyt et al., 2015) (Supplementary Data S1).

### 2.4 Convolutional neural network model development

This study investigated the addition of DeepSurv architecture to four deep learning frameworks (InceptionResNetV2, ResNet50, InceptionV3, and VGG19). These object recognition models were pretrained on ImageNet data. Using Keras pre-processing image, the input images were standardized to at least 300 × 300 pixels before being normalized to 336 × 336 pixels. Transfer learning includes using convolutional kernels to extract features from various layers, with a regularization dropout rate of 0.2. Training used a batch size of 16 and 300 epochs each iteration, with callback functions optimizing the dynamic learning rate.

### 2.5 DeepSurv model architecture and hyperparameter tuning

The Cox Proportional Hazards (CPH) model estimates the true hazard function $h\left(x\right)$ via a linear function $\hat{h_{\beta }}\left(x\right)=\beta^{T}x$ . To perform Cox regression, weights are adjusted to optimize the Cox partial likelihood, which is the product of the probabilities of each individual’s event occurring at their respective time. The CPH maximum partial likelihood function, parameterized by β , is defined as:

$$L_{c}\left(\beta \right)=\prod_{{i:E}_{i}=1}\frac{\exp\;\left(\hat{h_{\beta }}\left(x_{i}\right)\right)}{\sum_{j\epsilon R\left(T_{i}\right)}\exp\;\left(\hat{h_{\beta }}\left(x_{i}\right)\right)}.$$ (1)

Eq. 1: The Cox partial likelihood is optimized by the weights β , which reflect the effect of the baseline covariates x on the hazard function. The numerator of the partial likelihood is the risk score of patient i at their conception time $T_{i}$ ​, and the denominator is the sum of the risk scores of all the patients who are still have not conceived at $T_{i}$ ​. $E_{i}$ is the event indicator, which is 1 if patient i conceived and 0 otherwise. $x_{i}$ ​ is the vector of baseline covariates for patient i .

DeepSurv refine the modified Cox partial likelihood, which replace the linear combination of features $\hat{h_{\beta }}\left(x\right)$ with the output of a network $\hat{h_{\theta }}\left(x\right)$ . The output of the network is a single node that estimates the risk function $\hat{h_{\theta }}\left(x\right)$ parameterized by the network’s weight parameters θ. The loss function were set to be the negative log-likelihood, given by (22):

$$l_{\theta }=-\frac{1}{N_{E=1}}\sum_{i:E_{i}=1}\left({\hat{h}}_{\theta }\left(x_{i}-\log\sum_{j\in R\left(T_{i}\right)}e^{{\hat{h}}_{\theta }\left(x_{j}\right)}\right)+\lambda ∙{\left|\left|\theta \right|\right|}_{2}^{2}\right).$$ (2)

Eq. 2: The number of patients with observable events is denoted by $N_{E=1}$ ​, and λ is a regularization parameter that controls the trade-off between model complexity and data fit.

Through an exhaustive grid search (Supplementary Figure S1), we fine-tuned the model’s hyperparameters, including the number of layers (2 hidden layers), number of nodes (8 per layer), activation function (scaled exponential linear unit; SeLU), learning rate (0.154), weight decay (0.00567), momentum (0.887), dropout rate (0.500), and optimizer (Nadam). Moreover, DeepSurv employed batch normalization, stochastic gradient descent with Nesterov momentum, gradient clipping, and learning rate schedules. 1,000 epochs were used to obtain well-trained weights. The model was saved as an executable file (.exe) and uploaded to the GitHub repository (Proportional, 2022). The models block diagram is shown in Figure 1C.

### 2.6 Model evaluation

The performance of the model was assessed using two metrics. The time-ROC curve (TimeROC package in R, version 3.6.2) was used to evaluate accuracy at various time points. The c-index (Survcomp package in R, version 3.6.2) was used to compare the predicted conception time to the actual conception time. Parameters, floating-point operations (FLOPs), and average inference time were used to evaluate the computational complexity of the model, including memory consumption, computational cost, and efficiency, respectively. The number of parameters reflects the memory required to store the model weights. The FLOPs measure the number of arithmetic operations needed to perform a forward pass of the model. The average inference time is the average duration of a single prediction on a new image.

### 2.7 Statistical analysis

Patient demographics and clinical characteristics were summarized using descriptive statistics. The Wilcoxon rank-sum test compared the continuous variables between groups, whereas the Chi-squared test assessed categorical variables. Cox proportional hazard regression was used to calculate HR and evaluate the ART benefits among risk groups. The statistical significance was set at *p* < 0.05.

### 2.8 Subfertility risk visualization

Patient demographics and clinical characteristics were summarized using descriptive statistics. The Wilcoxon rank-sum test compared the continuous variables between groups, whereas the Chi-squared test assessed categorical variables. Cox proportional hazard regression was used to calculate HR and evaluate the ART benefits among risk groups. The statistical significance was set at *p* < 0.05 (Selvaraju et al., 2020).

### 2.9 Ethical approval

The Research Ethics Committee of Beijing Obstetrics and Gynecology Hospital approved and supervised the study. The revised version was approved by the Chinese Ethics Committee of Registering Clinical Trials (protocol number IEC-B-03-v01-FJ1 and ChiECRCT20220180).

### 2.10 Sample size

The sample size was calculated to ensure test efficacy, achieving a desired power of 90% at a significance level of 0.05 (Supplementary Figure S2).

## 3 Results

### 3.1 Patients characteristics

Table 1 shows the baseline of patients. Among the study participants, 13.3% were excluded for various reasons, such as divorce, contraception, or the postponement of reproductive plans. As a result, the training, validation, and testing sets contained 303, 76, and 102 patients with conception outcomes, respectively. Upon follow-up, the percentages of patients who achieved ongoing pregnancy in the training, validation, and testing sets were 40.0% (112/303), 47.4% (36/76), and 38.2% (39/102), respectively. There were no statistically significant differences in clinical indicators such as age, the severity of IUAs (as measured by AFS and CSGE scores), and the rates of successful pregnancy within 1 year and during the follow-up period between the three patient groups.

**TABLE 1:** Overview of the demographics and other characteristics of the recruited patients.

Characteristics		Modelling cohort	Test cohort	*p* ^a^
Age [n (%)]	<35 y	302 (70.2%)	85 (68%)	0.66
	≥35 y	128 (29.8%)	40 (32%)	
Symptom duration [Months, Median (quartile)]		23 (8, 36)	15 (10, 36)	0.94
Menstrual pattern^b^ [n (%)]	**Normal**	39 (9.1%)	10 (8%)	0.97
	**<1/2**	132 (30.7%)	37 (29.6%)	
	**Hypomenorrhea**	224 (52.1%)	67 (53.6%)	
	**Amenorrhea**	35 (8.1%)	11 (8.8%)	
Age at menarche [Median (quartile)]		13 (12, 14)	13 (12, 14)	0.45
Menstrual volume before endometrial injury	**Heavy**	19	5	0.8
	**Normal**	411	100	
Gravidity [Median (quartile)]		2 (1, 3)	2 (1, 3)	0.11
Parity [Median (quartile)]		0 (0, 0)	0 (0, 0)	0.28
Missed abortion [Median (quartile)]		0 (0, 1)	0 (0, 1)	0.46
Cesarean delivery [Median (quartile)]		0 (0, 0)	0 (0, 0)	0.28
Uterine aspiration [Median (quartile)]		1 (0, 2)	1 (0, 2)	0.32
Medication abortion [Median (quartile)]		0 (0, 0)	0 (0, 0)	0.53
Spontaneous abortion [Median (quartile)]		0 (0, 0)	0 (0, 0)	0.75
Dilation and evacuation [Median (quartile)]		0 (0, 1)	0 (0, 1)	0.56
Uterine volume [Median (quartile)]		31.38 (25.1, 41.84)	31.38 (25.1, 41.84)	0.95
BMI [kg/m^2^, Mean (SD)]		23.22 (0.67)	22.16 (0.27)	0.4
AFS [n (%)]	**Mild (1-4)**	7 (1.6%)	4 (3.2%)	0.527
	**5-8**	260 (60.5%)	73 (58.4%)	
	**Severe (9-12)**	163 (37.9%)	48 (38.4%)	
CSGE [n (%)]	**Mild (0-8)**	29 (6.7%)	16 (12.8%)	0.064
	**Moderate (9-18)**	369 (85.8%)	103 (82.4%)	
	**Severe (19-28)**	32 (7.4%)	6 (4.8%)	
Ongoing pregnancy [n (%)]		148 (39.5%)	39 (38.24%)	0.91
IVF [n (%)]		62 (16.4%)	12 (11.8%)	0.283
Unexpected exclusion [n (%)]		16 (3.72%)	9 (7.2%)	0.15
Missed visit [n (%)]		35 (8.45%)	14 (12.07%)	0.28

^a^ Comparison between groups was performed by chi-square, Wilcox, and *t*-test, respectively.

^b^ In comparison with the change in the menstrual pattern before the IUA.

### 3.2 Model development and performance in modelling cohart

**FIGURE 2:**
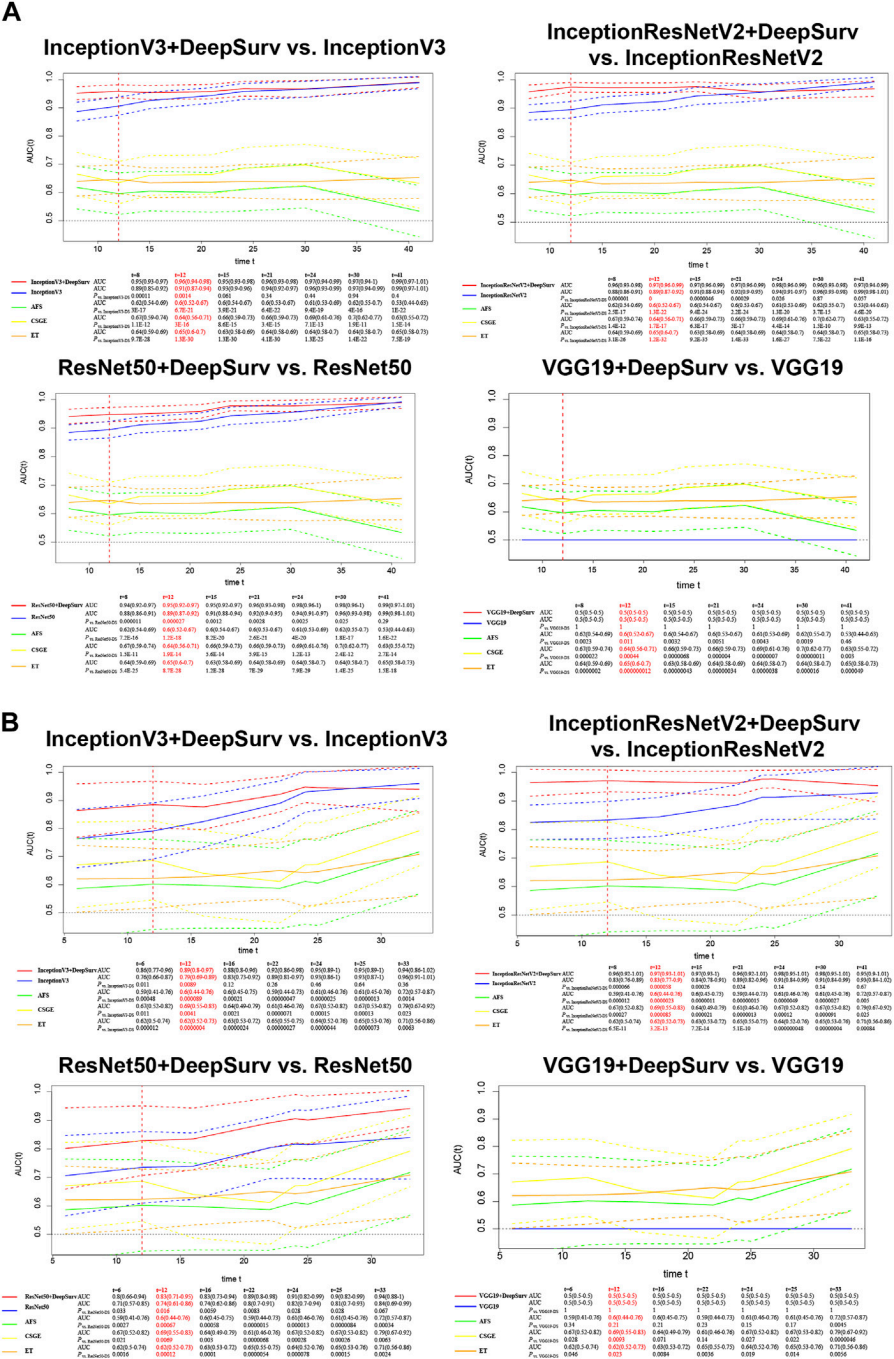
Time-dependent AUCs of InceptionV3+DeepSurv, InceptionResNetV2+DeepSurv, ResNet50+DeepSurv, and VGG19+DeepSurv models are shown for the training **(A)** and validation **(B)** sets. The *x*-axis represents the duration of time, and the *y*-axis represents the area under the curve (AUC) of the receiver operating characteristic (ROC) curve. The plot demonstrates the performance of the models over time for each dataset.

We calculated the time-dependent AUCs for both the training and validation sets after training on the training set to achieve the best weights (see the loss gradient curve in the Supplementary Figure S3). In the case of InceptionV3+DeepSurv, the AUC for the training set was 0.96 (95% CI: 0.94–0.98) and 0.89 (95% CI: 0.8–0.97) for the validation set. InceptionResNetV2+DeepSurv achieved an AUC of 0.97 (95% CI: 0.96–0.99) for the training set and 0.97 (95% CI: 0.93–1.01) for the validation set. ResNet50+DeepSurv had an AUC of 0.95 (95% CI: 0.92–0.97) for the training set and 0.83 (95% CI: 0.71–0.95) for the validation set when predicting natural conception within 1 year. These models with the DeepSurv architecture significantly outperformed those without it (*p* < 0.05, Figure 2). Notably, models based on the VGG19 framework continuously performed poorly, regardless of the presence of the DeepSurv architecture.

### 3.3 Testing models on the test set

**FIGURE 3:**
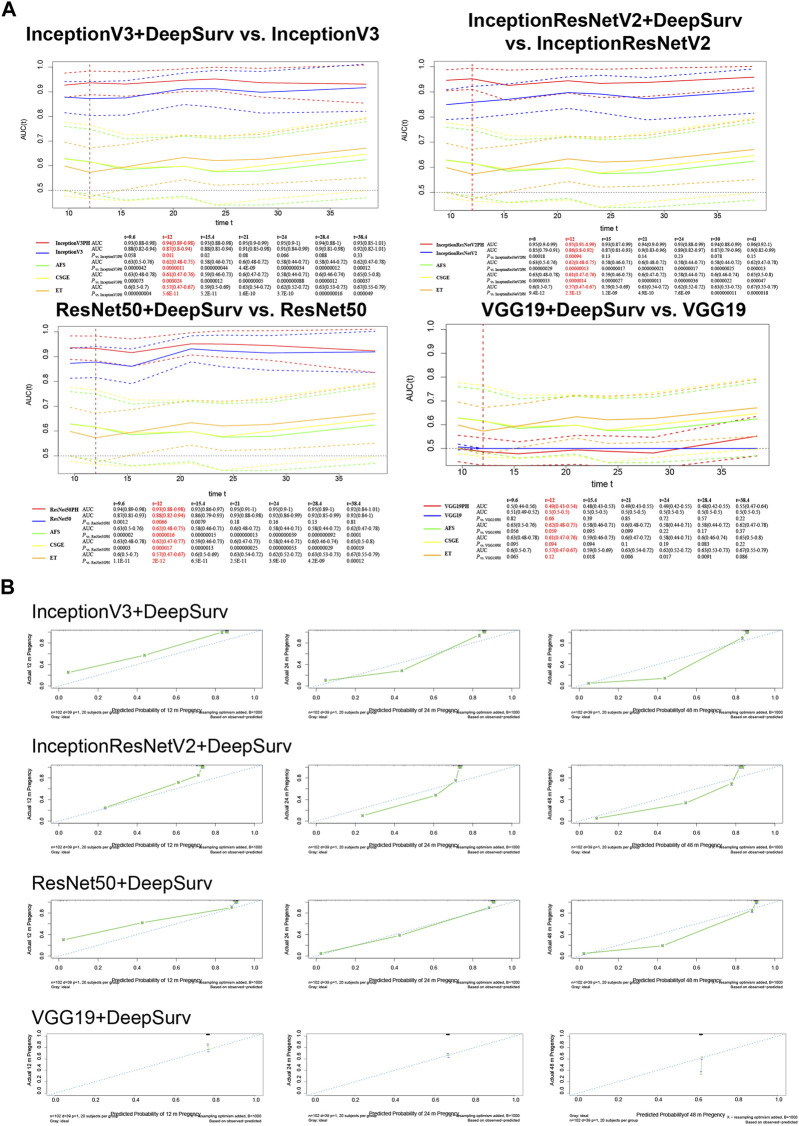
External validation on the test set of InceptionV3+DeepSurv, InceptionResNetV2+DeepSurv, ResNet50+DeepSurv and VGG19+DeepSurv by **(A)** time-dependent AUCs and **(B)** Calibration plots over 12, 24, and 48 months. The calibration plots are presented for each model separately, with the *x*-axis showing predicted probabilities and the *y*-axis showing observed frequencies. The plot provides a measure of how well the predicted probabilities match the observed frequencies.

The models underwent external validation on the test set, and for predicting natural conception within 1 year, InceptionV3+DeepSurv (AUC = 0.94, 95% CI; 0.89–0.98), InceptionResNetV2+DeepSurv (AUC = 0.95, 95% CI; 0.91–0.99), and ResNet50+DeepSurv (AUC = 0.93, 95% CI; 0.88–0.98) significantly outperformed InceptionV3 (AUC = 0.87, 95% CI; 0.8–0.94), InceptionResNetV2 (AUC = 0.86, 95% CI; 0.8–0.92), and ResNet50 (AUC = 0.88, 95% CI; 0.82–0.94), with *p*-values equal to 0.011, 9.4 × 10^−4^, and 6.6 × 10^−3^, respectively. Furthermore, the models above still revealed significant improvements when comparing clinical markers such as AFS, CSGE, and endometrial thickness (Figure 3A).

The calibration plot illustrates the calibration deviance and the model’s predictions compared to actual events (Figure 3B). Overall, the actual conception outcomes of IUAs at 12, 24, and 48 months closely matched the 45° line, demonstrating a high degree of consistency. There was no significant difference among the three models. The predictions obtained by these models were significantly superior to the clinical indicators such as AFS, CSGE, and endometrial thickness (Table 2).

**TABLE 2:** Comparison of concordance indexes for each assessment method.

Terms	c-index (95%CI)	*p* vs. InceptionV3+DeepSurv	*p* vs. InceptionResNetV2+DeepSurv	*p* vs. ResNet50+DeepSurv	*p* vs. VGG19+DeepSurv	*p* vs. AFS	*p* vs. CSGE
InceptionV3+DeepSurv	0.9 (0.86–0.93)	—	—	—	—	—	—
InceptionResNetV2+DeepSurv	0.89 (0.86–0.93)	0.6	—	—	—	—	—
ResNet50+DeepSurv	0.9 (0.86–0.94)	0.66	0.57	—	—	—	—
VGG19+DeepSurv	0.56 (0.31–0.81)	0.0048	0.0048	0.0048	—	—	—
AFS	0.61 (0.49–0.73)	1.27 × 10^−06^	1.07 × 10^−06^	1.21 × 10^−06^	0.65	—	—
CSGE	0.6 (0.49–0.71)	5.70 × 10^−07^	5.02 × 10^−07^	5.16 × 10^−07^	0.63	0.4	—
Endometrial thickness	0.77 (0.61–0.93)	0.05	0.047	0.046	0.93	0.97	0.97

### 3.4 Model region of interest visualization

**FIGURE 4:**
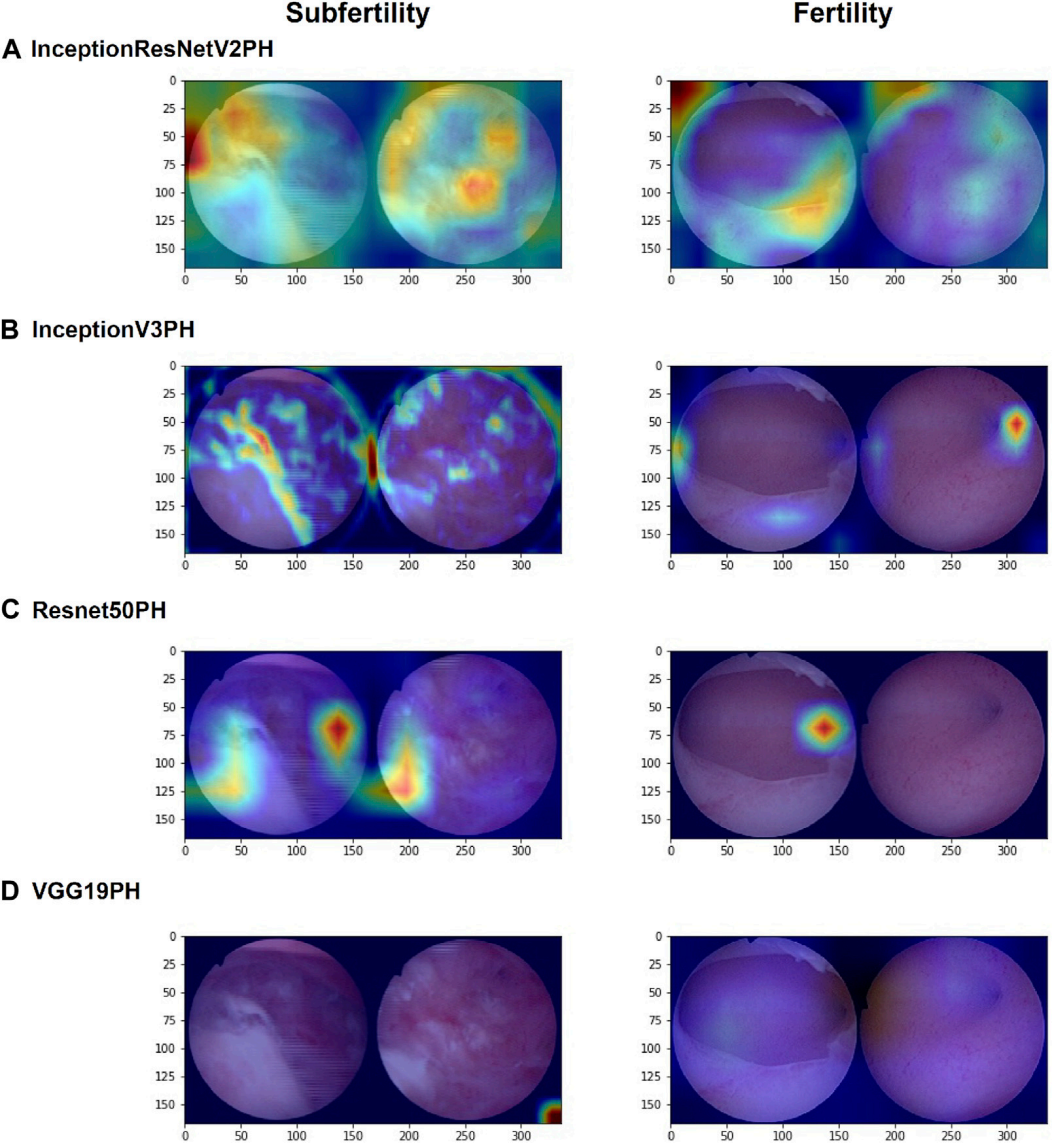
Grad-CAM visualizations of the regions of interest (ROIs) for InceptionResNetV2+DeepSurv, **(A)** InceptionV3+DeepSurv, **(B)** ResNet50+DeepSurv, **(C)** and VGG19+DeepSurv **(D)** models are presented. The figure shows the results for patients who were not pregnant during postoperative follow-up on the left and for patients who had successful pregnancies after surgery on the right. The visualization highlights the regions of the image that were most important for the model’s prediction.

The use of Grad-CAM facilitates the visualization of the ROI of the model for images during machine learning (Figure 4). The ROI analysis indicates that InceptionResNetV2+DeepSurv, InceptionV3+DeepSurv, and ResNet50+DeepSurv all exhibit highlighted regions presenting image attributes on which the machine learning classification is based. Conversely, VGG19+DeepSurv lacks a comparable feature display, suggesting that this model may struggle with extracting image features from hysteroscopic images that can be used for classification. Furthermore, the first three models have slightly different understandings of image attributes. InceptionResNetV2+DeepSurv focuses more on intrauterine morphology; InceptionV3+DeepSurv focuses on the state of the endometrium and the fallopian tube ostias, while ResNet50+DeepSurv gives attention to the intrauterine morphology and the cornua. Nonetheless, the three machine learning models are consistent with the clinical understanding of the condition. It may be possible to use these models in clinically available applications by integrating them with the ROI display.

### 3.5 Models’ computation complexity and application deployment

Table 3 presents a comparison of the computational complexity of the four models utilized in this research. The evaluation of these models includes parameters such as the number of parameters, FLOPs, average inference time, and model performance metrics (c-index, AUC at 1 year, and AUC at 2 years). In general, the InceptionV3, InceptionResNetV2, and ResNet50 models integrated with the DeepSurv framework exhibit similar performance characteristics. Specifically, the InceptionV3+DeepSurv model demonstrates a slightly lower count of parameters and FLOPs compared to the ResNet50+DeepSurv model, yet it delivers superior performance. Conversely, the InceptionResNetV2+DeepSurv model boasts the highest number of parameters and FLOPs among the models. On the other hand, the VGG19+DeepSurv model features the lowest count of parameters and FLOPs, correlating with the lowest performance metrics. Overall, the InceptionV3+DeepSurv model emerges as the top-performing model in terms of accuracy and computational complexity. As a viable option, the ResNet50+DeepSurv model offers a comparable performance level and computational efficiency compared to the InceptionV3+DeepSurv model.

**TABLE 3:** Computational complexity of models.

Models	Params^a^	FLOPs^b^	avg_time^c^	c-index	AUC (1year)	AUC (2years)
InceptionResNetV2+DeepSurv	54,336,736	14,094,325,732	0.2145	0.89 (0.86–0.93)	0.95 (0.91–0.99)	0.93 (0.88–0.99)
ResNet50+DeepSurv	23,587,712	9,210,755,721	0.0696	0.9 (0.86–0.94)	0.93 (0.88–0.98)	0.95 (0.9–1)
InceptionV3+DeepSurv	21,802,784	6,234,156,566	0.0555	0.9 (0.86–0.93)	0.94 (0.89–0.98)	0.95 (0.9–1)
VGG19+DeepSurv	20,024,384	43,759,363,472	0.0452	0.56 (0.31–0.81)	0.49 (0.43–0.54)	0.49 (0.42–0.55)

^a^ Params: parameters.

^b^ FLOPs: floating-point operations.

^c^ Avg_time: average inference time.

The application we developed using the InceptionV3+DeepSurv model as an example is user-friendly and can operate efficiently without requiring advanced coding skills (Video). The red highlighted areas on the images visually reflect the high-risk factors for subfertility. We also assessed the probability of successful pregnancy for the patients within one and 2 years. This model estimates the patient’s infertility risk, as demonstrated in the two examples. This individualized risk assessment is critical for postoperative patients undergoing assisted reproductive technologies. By identifying the subfertility risk factors through the model, healthcare professionals can focus on infertility risk factors and build individualized treatment options.

### 3.6 DeepSurv architecture models for fertility prognosis stratification in ART

We categorized patients based on the fertility prognosis predicted by the previously mentioned code-free AI application. The ART benefit rate was significantly higher among individuals with a low likelihood of natural conception within 1 year, with an HR of 3.13 (95% CI: 1.22–8.02, *p* = 0.017), compared to those with a high likelihood of natural conception within 1 year, where the HR was 0.52 (95% CI: 0.24–1.14, *p* = 0.101). The model’s stratification for ART benefit outperformed AFS, CSGE, and endometrial thickness (Table 4).

**TABLE 4:** Hazard ratio of model’s stratification for natural conception within 1 year.

Models			ART benefit HR (95%CI)	*p*-Value
PH AI application	Conception probability	<0.5	3.13 (1.22–8.02)	0.017
		>0.5	0.52 (0.24–1.14)	0.101
AFS	≥5		1.644 (0.943–2.864)	0.0794
	<5		0.522 (0.065–4.193)	0.541
CSGE	≥18		1.11 (0.134–9.235)	0.922
	<18		1.53 (0.774–3.026)	0.222
Endometrial thickness	≥7 mm		1.259 (0.696–2.28)	0.447
	<7 mm		2.272 (0.265–19.45)	0.454

## 4 Discussion

Selecting an appropriate deep learning technique for fertility assessment is relevant in effectively diagnosing and treating IUAs, a disease characterized by endometrial damage. To the best of our knowledge, this is the first study that applied deep learning models to the conception prediction and management task using hysteroscopic images, and compared their performance and complexity using the DeepSurv framework. Various deep learning algorithms were explored to identify a CNN model suited for analyzing hysteroscopic images, which was then integrated with CPH neural networks to evaluate the subfertility risks and predict the probabilities of conception at different times and assist in formulating optimal treatment strategies after hysteroscopic adhesiolysis for IUAs.

Traditional approaches for evaluating IUAs primarily rely on clinical scoring systems, including AFS, ESGE, and CSGE. These scoring systems aim to clinically assess the severity of IUAs and predict pregnancy outcomes (Cao et al., 2021). Cao et al. (2021) conducted a retrospective study to investigate the effectiveness of different AS evaluation systems in determining reproductive outcomes. AUCs for AFS and CSGE were 0.663 and 0.684, respectively, consistent with our findings. Variables in the AFS scoring system include the extent and nature of adhesions and the patient’s menstrual status. However, these approaches are subjective and may not accurately reflect the patient’s endometrial function (AAGL, 2010).

A few studies have explored the application of artificial intelligence algorithms in the field of IUAs. In our previous study, we used machine learning algorithms such as decision trees and XGboost to predict the pregnancy outcomes of patients based on clinical data (Li et al., 2022; Zhu et al., 2022; Li et al., 2023a). Zhao et al. (2022) also developed a logistic prediction model using clinical parameters such as age, preoperative AFS score, preoperative uterine cavity length, and visibility of bilateral fallopian tube ostia during hysteroscopy review. These methods achieved excellent predictive performance, with AUCs of 0.8–0.9. However, these studies also acknowledged the limitations of their research, such as the lack of consensus and consistency on the clinical indicators, such as the IUA scoring system. Sun et al. (2024) proposed a logistic prediction model based on various evaluation parameters of 3D transvaginal ultrasound, which also showed promising results, but suffered from the same drawbacks of subjectivity and variability. The subjective differences in judgment and evaluation among different doctors are important factors that hinder the wide application of these models in clinical practice.

Our study’s innovation lies in applying image deep learning technology for automated analysis. This approach improves the objectivity of the results while reducing the assessment effort for clinical physicians. In the validation and test sets, our model achieved accuracies of AUC of 0.89 and 0.94, respectively, outperforming traditional clinical assessment indicators.

Postoperative hysteroscopy second-look images were selected as the training dataset for the model in this study. Both hysteroscopy and ultrasound are routinely used diagnostic tools for assessing the fertility of a patient and the condition of their uterus, and each has advantages. Hysteroscopy is a direct intrauterine visualization technique that assesses endometrial status and endometrial vascular distribution to measure endometrial receptivity, with positive results reported in limited studies. The sensitivities of endometrial status and endometrial vascular distribution were 75% and 71.43%, whereas the specificities were 71.43% and 61.11%, respectively (Craciunas et al., 2019). Using image deep learning analysis techniques, the present study further improved the accuracy of its predictive outcomes. Although ultrasound comprehensively evaluates the uterus and measures endometrial thickness, it has difficulty visualizing intrauterine pathology. Ultrasound possesses a high sensitivity but a low specificity of around 3%. However, previous research has demonstrated that the clinical information presented by second-look hysteroscopy is more conducive to predicting pregnancy outcomes than preoperative hysteroscopy examinations.

The present study investigates the hypothesis that introducing a DeepSurv framework into image-based deep learning networks can improve clinical practice. Clinical decision-making in managing fertility in patients with postoperative IUA often focuses on two major factors: a patient’s risk of infertility and the duration of postoperative infertility. Patients with difficulty conceiving within 1 year after surgery or with severe adhesions are usually referred to ART treatments.

This study augments the transfer learning model architecture with the DeepSurv architecture, which extends the application of the CPH model using a neural network architecture to incorporate nonlinear parameters, such as image features. This has significant implications for clinical decision-making and patient counseling. The adoption of the CPH neural network architecture provides several advantages over conventional transfer learning models for medical image classification (Li et al., 2023b; Zhao et al., 2023). Firstly, it enables the prediction of time-to-event outcomes, such as the probability of conception over time. This capability allows for more accurate medical prognosis stratification and the calculation of cumulative risk for fertility outcomes.

The CPH deep neural network is proficient in characterizing the influence of covariates on the hazard function, accommodating the immediate risk of failure. This deviates from conventional deep learning classifiers, as the CPH neural network facilitates the computation of fertility outcomes, hazard functions, and hazard ratios across diverse patient cohorts. In contrast to regular deep learning classifiers that output probabilities, the DeepSurv captures temporal dynamics and relative risks of outcomes.

Previous studies have demonstrated the effectiveness of DeepSurv in facilitating risk stratification and treatment recommendations for various disorders, such as non-small cell lung cancer and head and neck cancer (Howard et al., 2020; She et al., 2020; Liu et al., 2022). Building on these findings, the integration of the two network frameworks allows us to transform the convolutional neural network output into a continuous hazard ratio, taking into account both fertility outcomes and time to conception. This integration provides a robust risk stratification function and the ability to calculate cumulative time risk for risk scoring.

As evidenced by our results, the model augmented with the DeepSurv architecture significantly outperforms the transfer learning model performing classification alone in predicting the probability of conception at different time points, particularly within the first year. Conforming to clinical settings, the DeepSurv model of our study accurately predicts subfertility risk and time to conception, outperforming other methods and conventional clinical indicators.

In this study, we applied four deep learning models, namely, InceptionV3, InceptionResNetV2, ResNet50, and VGG19, combined with the DeepSurv framework, to predict the conception probability and management in IUA following hysteroscopic adhesiolysis. Our results showed that the InceptionV3+DeepSurv model achieved the best performance in terms of accuracy and computational efficiency, followed by the ResNet50+DeepSurv model. The InceptionResNetV2+DeepSurv model had the highest complexity and the VGG19+DeepSurv model had the lowest performance among the models.

InceptionV3 and InceptionResNetV2 are based on Inception modules, and ResNet50 is based on the idea of residual connections, which all allow the network to learn the identity function and avoid the degradation problem when the network depth increases (Jain et al., 2021). However, they differ in the way they implement the inception modules, which are designed to capture multi-scale features and reduce the number of parameters. The InceptionV3 model uses a combination of 1 × 1, 3 × 3, and 5 × 5 convolutions, as well as 3 × 3 max pooling, to form the inception modules (Szegedy et al., 2016). The InceptionResNetV2 model adds residual connections to the inception modules, and uses a more efficient factorization of the convolutions (Szegedy et al., 2017). The ResNet50 model uses a simpler structure of 1 × 1, 3 × 3, and 1 × 1 convolutions, followed by batch normalization and ReLU activation, to form the residual blocks (He et al., 2016).

Based on our results, the InceptionV3 and Resnet50 model combined with DeepSurv has a better balance between complexity and performance, and can capture more relevant features from the hysteroscopic images for the conception prediction task. However, the VGG19+DeepSurv model had the lowest complexity and performance among the models, which may be due to the fact that the VGG19 model does not use any residual connections or inception modules, and relies on a large number of fully connected layers, which are prone to overfitting and have a high computational cost (Jain et al., 2021).

Hysteroscopic images necessitate evaluating intrauterine morphology and microscopic characteristics, such as endothelial vascularity and glandular conditions, which are reflected in the final ROI presentation and provide valuable clinical insights. Previous research identified associations between the reproductive prognosis for IUAs and factors like intrauterine morphology, tubal ostia status, endometrial thickness, and endometrial blood supply (Zhu et al., 2022). InceptionResNetV2, ResNet50, and InceptionV3 effectively capture these traits, resulting in variations in final ROIs. These visual findings are also integrated into the output interface of the AI program.

Although image deep learning has many advantages, its clinical applications are still rare due to its laboratory stage. The main focus of the study should be on how to make these models applicable to clinicians without any coding experience. The ROI visualization and the DeepSurv model are combined in this study to assess the probability of pregnancy for patients within one or 2 years. The model helps visualize the patient’s fertility and detect potential intrauterine factors that may affect pregnancy. Similar to clinical scoring systems like AFS for evaluating IUAs, predicting a patient’s prognosis has clinical relevance in determining the need for postoperative intervention.

**FIGURE 5:**
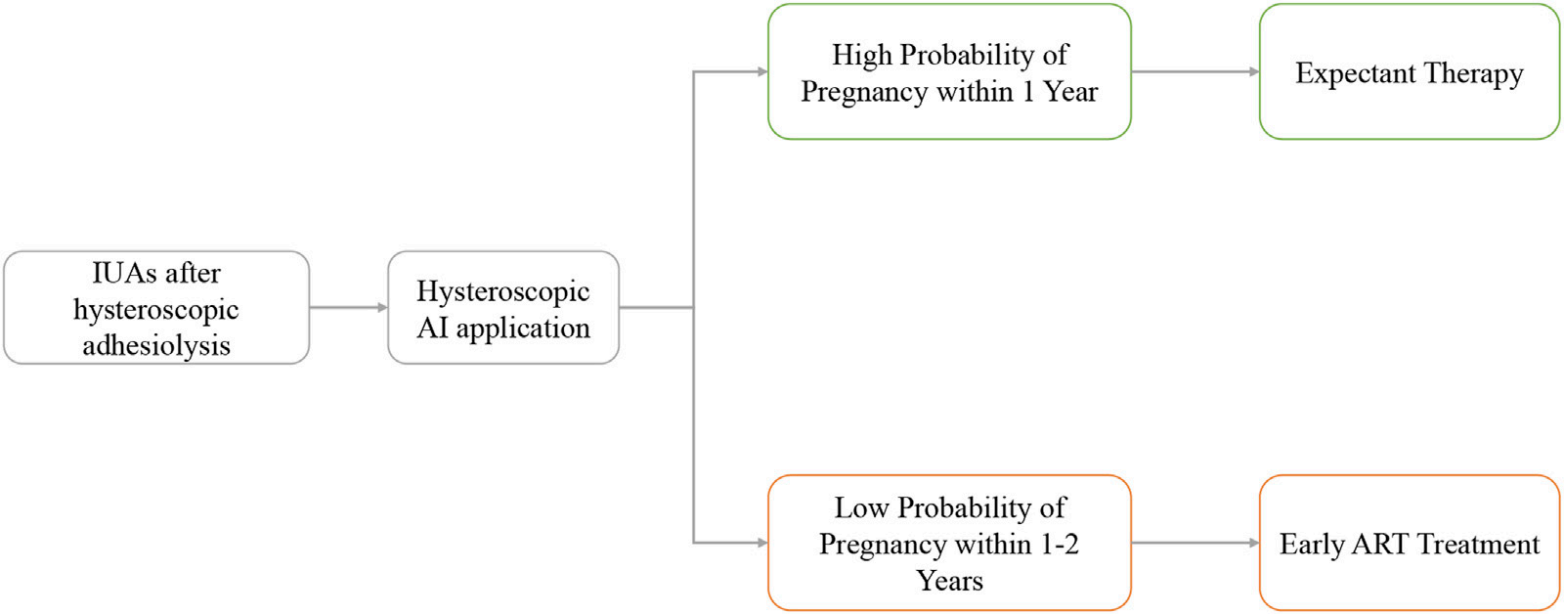
Specific management strategy flow of the AI application.

Further stratified analysis revealed that patients with a low probability of natural pregnancy within 1 year significantly benefit from ART interventions. This encourages early ART intervention for such patients, reducing time and treatment costs. The specific management strategy is presented in Figure 5. Furthermore, experimental treatments, such as stem cells and amnion grafts (Gan et al., 2017), can be targeted for treating refractory IUAs based on prognostic stratification, optimizing the allocation of medical resources.

This study explored the prediction model of fertility prognosis based on hysteroscopic images of IUA, and conducted a comprehensive analysis and evaluation of the accuracy and computational complexity of various models. This study provided promising results; however, some limitations must be acknowledged. The issue of small sample size is a common challenge for image-deep-learning models, which may affect the reliability and generalizability of the results. Therefore, larger sample sizes are required to ensure that the models trained are applicable in real-world settings. We plan to further increase the sample size beyond this pilot study to validate the robustness of the model. Moreover, our future goal is to use the prospective database and standardize the image acquisition process to increase the model’s generalization performance. The results demonstrate that the final model performs favorably across all three randomly assigned datasets. In future research, we will explore more advanced deep learning techniques and conduct prospective studies to further validate the models, along with larger datasets to make predictive models more accurate and applicable. Additionally, combining other clinical and imaging data for multi-modal learning is also a potential direction worth exploring.

## 5 Conclusion

This study investigates the prediction capabilities of four commonly used image deep learning transfer models and their integration with the DeepSurv framework for assessing fertility risks. Among these models, InceptionResNetV2+DeepSurv, ResNet50+DeepSurv, and InceptionV3+DeepSurv perform well in extracting features from hysteroscopic images and providing a multi-faceted perspective on such characteristics in IUAs. Based on these findings, the models are used in a code-free application program to highlight abnormal intrauterine areas, predict the probability of pregnancy within one and 2 years after surgery, stratify fertility levels, and assist clinical decision-making in developing individualized postoperative fertility management plans.

## Data Availability

The raw data supporting the conclusion of this article will be made available by the authors, without undue reservation.

## Supplementary material

SUPPLEMENTARY VIDEO S1 Demonstration of the AI application automatic analysis of hysteroscopic images.

## References

[B1] AAGL (2010). AAGL practice report: practice guidelines for management of intrauterine synechiae. J. Minim. Invasive Gynecol. 17 (1), 1–7. 10.1016/j.jmig.2009.10.009 20129325

[B2] Bossuyt P. M. Reitsma J. B. Bruns D. E. Gatsonis C. A. Glasziou P. P. Irwig L. (2015). STARD 2015: an updated list of essential items for reporting diagnostic accuracy studies. BMJ 351, h5527. 10.1136/bmj.h5527 26511519 PMC4623764

[B3] Bosteels J. van Wessel S. Weyers S. Broekmans F. J. D'Hooghe T. M. Bongers M. Y. (2018). Hysteroscopy for treating subfertility associated with suspected major uterine cavity abnormalities. Cochrane Database Syst. Rev. 12 (12), Cd009461. 10.1002/14651858.cd009461.pub4 30521679 PMC6517267

[B4] Cao M. Pan Y. Zhang Q. You D. Feng S. Liu Z. (2021). Predictive value of live birth rate based on different intrauterine adhesion evaluation systems following TCRA. Reprod. Biol. Endocrinol. 19 (1), 13. 10.1186/s12958-021-00697-1 33482838 PMC7821669

[B5] Craciunas L. Gallos I. Chu J. Bourne T. Quenby S. Brosens J. J. (2019). Conventional and modern markers of endometrial receptivity: a systematic review and meta-analysis. Hum. Reprod. Update 25 (2), 202–223. 10.1093/humupd/dmy044 30624659

[B6] Esteva A. Robicquet A. Ramsundar B. Kuleshov V. DePristo M. Chou K. (2019). A guide to deep learning in healthcare. Nat. Med. 25 (1), 24–29. 10.1038/s41591-018-0316-z 30617335

[B7] Gan L. Duan H. Sun F.-Q. Xu Q. Tang Y.-Q. Wang S. (2017). Efficacy of freeze-dried amnion graft following hysteroscopic adhesiolysis of severe intrauterine adhesions. Int. J. Gynaecol. Obstet. 137 (2), 116–122. 10.1002/ijgo.12112 28170094

[B8] Hanstede M. M. F. van der Meij E. Veersema S. Emanuel M. H. (2021). Live births after Asherman syndrome treatment. Fertil. Steril. 116 (4), 1181–1187. 10.1016/j.fertnstert.2021.05.099 34130799

[B9] He K. Zhang X. Ren S. Sun J. (2016). “Deep residual learning for image recognition,” in Proceedings of the IEEE conference on computer vision and pattern recognition, USA, 17-19 June 1997. (IEEE).

[B10] Hooker A. B. Lemmers M. Thurkow A. L. Heymans M. W. Opmeer B. C. Brölmann H. A. (2014). Systematic review and meta-analysis of intrauterine adhesions after miscarriage: prevalence, risk factors and long-term reproductive outcome. Hum. Reprod. Update 20 (2), 262–278. 10.1093/humupd/dmt045 24082042

[B11] Howard F. M. Kochanny S. Koshy M. Spiotto M. Pearson A. T. (2020). Machine learning-guided adjuvant treatment of head and neck cancer. JAMA Netw. Open 3 (11), e2025881. 10.1001/jamanetworkopen.2020.25881 33211108 PMC7677764

[B12] Jain S. Singhania U. Tripathy B. Nasr E. A. Aboudaif M. K. Kamrani A. K. (2021). Deep learning-based transfer learning for classification of skin cancer. Sensors Basel, Switz. 21 (23), 8142. 10.3390/s21238142 PMC8662405 34884146

[B13] Katzman J. L. Shaham U. Cloninger A. Bates J. Jiang T. Kluger Y. (2018). DeepSurv: personalized treatment recommender system using a Cox proportional hazards deep neural network. BMC Med. Res. Methodol. 18 (1), 24. 10.1186/s12874-018-0482-1 29482517 PMC5828433

[B35] Li B. Chen H. Lin X. Duan H. (2024). Multimodal Learning system integrating electronic medical records and hysteroscopic images for reproductive outcome prediction and risk stratification of endometrial injury: A multicenter diagnostic study. Int J Surg. In Press. 10.1097/JS9.0000000000001241 PMC11175765 38935827

[B14] Li B. Duan H. Wang S. Wu J. Li Y. (2022). Gradient boosting machine learning model for defective endometrial receptivity prediction by macrophage-endometrium interaction modules. Front. Immunol. 13, 842607. 10.3389/fimmu.2022.842607 35603216 PMC9120433

[B15] Li Y. Duan H. Wang S. (2023a). An XGBoost predictive model of ongoing pregnancy in patients following hysteroscopic adhesiolysis. Reprod. Biomed. Online 46 (6), 965–972. 10.1016/j.rbmo.2023.01.019 37037757

[B17] Li Y. Huang W. C. Song P. H. (2023b). A face image classification method of autistic children based on the two-phase transfer learning. Front. Psychol. 14, 1226470. 10.3389/fpsyg.2023.1226470 37720633 PMC10501480

[B18] Ling Y. Zhong J. Weng Z. Lin G. Liu C. Pan C. (2022). The prognostic value and molecular properties of tertiary lymphoid structures in oesophageal squamous cell carcinoma. Clin. Transl. Med. 12 (10), e1074. 10.1002/ctm2.1074 36245289 PMC9574489

[B19] Liu Z. Liu Y. Zhang W. Hong Y. Meng J. Wang J. (2022). Deep learning for prediction of hepatocellular carcinoma recurrence after resection or liver transplantation: a discovery and validation study. Hepatol. Int. 16 (3), 577–589. 10.1007/s12072-022-10321-y 35352293 PMC9174321

[B20] Proportional (2022). Proportional-hazard-architecture-transfer-networks. Available at: https://github.com/libohan1110/Proportional-Hazard-Architecture-Transfer-Networks.git.

[B21] Selvaraju R. R. Cogswell M. Das A. Vedantam R. Parikh D. Batra D. (2020). Grad-CAM: visual explanations from deep networks via gradient-based localization. Int. J. Comput. Vis. 128 (2), 336–359. 10.1007/s11263-019-01228-7

[B22] She Y. Jin Z. Wu J. Deng J. Zhang L. Su H. (2020). Development and validation of a deep learning model for non-small cell lung cancer survival. JAMA Netw. Open 3 (6), e205842. 10.1001/jamanetworkopen.2020.5842 32492161 PMC7272121

[B23] Smit J. G. Kasius J. C. Eijkemans M. J. C. Koks C. A. M. van Golde R. Nap A. W. (2016). Hysteroscopy before *in-vitro* fertilisation (inSIGHT): a multicentre, randomised controlled trial. Lancet 387 (10038), 2622–2629. 10.1016/s0140-6736(16)00231-2 27132052

[B24] Sun D. Yi S. Zeng F. Cheng W. Xu D. Zhao X. (2024). Developing and validating a prediction model of live birth in patients with moderate-to-severe intrauterine adhesions: a new approach with endometrial morphology measurement by 3D transvaginal ultrasound. Quantitative imaging Med. Surg. 14 (1), 995–1009. 10.21037/qims-23-1014 PMC10784096 38223019

[B25] Sutton R. T. Zai Ane O. R. Goebel R. Baumgart D. C. (2022). Artificial intelligence enabled automated diagnosis and grading of ulcerative colitis endoscopy images. Sci. Rep. 12 (1), 2748. 10.1038/s41598-022-06726-2 35177717 PMC8854553

[B26] Szegedy C. Ioffe S. Vanhoucke V. Alemi A. (2017). “Inception-v4, inception-resnet and the impact of residual connections on learning,” in Proceedings of the AAAI conference on artificial intelligence, USA, February 7–14, 2023. (IEEE).

[B27] Szegedy C. Vanhoucke V. Ioffe S. Shlens J. Wojna Z. (2016). “Rethinking the inception architecture for computer vision,” in Proceedings of the IEEE conference on computer vision and pattern recognition, USA, 17-19 June 1997. (IEEE).

[B28] Xu W. Zhang Y. Yang Y. Zhang S. Lin X. (2018). Effect of early second-look hysteroscopy on reproductive outcomes after hysteroscopic adhesiolysis in patients with intrauterine adhesion, a retrospective study in China. Int. J. Surg. 50, 49–54. 10.1016/j.ijsu.2017.11.040 29203342

[B29] Yu D. Li T. C. Xia E. Huang X. Liu Y. Peng X. (2008). Factors affecting reproductive outcome of hysteroscopic adhesiolysis for Asherman's syndrome. Fertil. Steril. 89 (3), 715–722. 10.1016/j.fertnstert.2007.03.070 17681324

[B30] Zhang Y. Wang Z. Zhang J. Wang C. Wang Y. Chen H. (2021). Deep learning model for classifying endometrial lesions. J. Transl. Med. 19 (1), 10. 10.1186/s12967-020-02660-x 33407588 PMC7788977

[B31] Zhao H. Zheng C. Zhang H. Rao M. Li Y. Fang D. (2023). Diagnosis of thyroid disease using deep convolutional neural network models applied to thyroid scintigraphy images: a multicenter study. Front. Endocrinol. (Lausanne). 14, 1224191. 10.3389/fendo.2023.1224191 37635985 PMC10453808

[B32] Zhao X. Gao B. Yang X. Zhang A. Jamail G. Li Y. (2021). The density of endometrial glandular openings: a novel variable to predict the live birth rate in patients with intrauterine adhesions following hysteroscopic adhesiolysis. Hum. Reprod. 36 (4), 965–975. 10.1093/humrep/deaa377 33486509 PMC7970727

[B33] Zhao X. Sun D. Zhang A. Huang H. Zhu X. Yi S. (2022). Uterine cavity parameters evaluated by hysteroscopy can predict the live birth rate for intrauterine adhesion patients. Front. Med. 9, 926754. 10.3389/fmed.2022.926754 PMC9249163 35783613

[B34] Zhu R. Duan H. Xu W. Wang S. Gan L. Xu Q. (2022). Decision tree model predicts live birth after surgery for moderate-to-severe intrauterine adhesions. BMC pregnancy childbirth 22 (1), 78. 10.1186/s12884-022-04375-x 35093014 PMC8801068

